# A Rare Diagnostic Dilemma of P‐ANCA/MPO Positive Crescentic Glomerulonephritis in an Immunosuppressed Lupus Patient

**DOI:** 10.1155/crin/1850355

**Published:** 2026-07-15

**Authors:** Omar Elrefy, Sweta Carpenter, Manu Saini, Manjula Balasubramanian, Daranee Chewaproug

**Affiliations:** ^1^ Jefferson Einstein Philadelphia Hospital, Department of Medicine, Division of Nephrology, Philadelphia, Pennsylvania, USA; ^2^ Department of Pathology, Jefferson Einstein Philadelphia Hospital, Philadelphia, Pennsylvania, USA

## Abstract

Perinuclear antineutrophil cytoplasmic antibodies (P‐ANCAs) and myeloperoxidase (MPO) antibodies are detected in 15%–25% of lupus nephritis patients, but systemic lupus erythematosus (SLE)/ANCA‐associated vasculitis (AAV) overlap syndrome is rare, occurring in approximately 2% of cases. We present a 57‐year‐old woman with SLE and antiphospholipid syndrome (APS) on belimumab, hydroxychloroquine, and prednisone, who presented with acute ischemic stroke requiring thrombectomy and rapidly progressive renal failure (creatinine rising from 1.1 to 5.2 mg/dL) with nephrotic‐range proteinuria (8.6 g/g). P‐ANCA titer was > 1:640 with MPO positivity, while anti‐dsDNA, C3, and C4 were normal. Kidney biopsy revealed crescentic glomerulonephritis with neutrophil‐rich infiltrates and immune complex deposits on electron microscopy but without “full house” immunofluorescence, favoring SLE/AAV overlap rather than isolated lupus nephritis flare. Treatment with methylprednisolone, rituximab, and anticoagulation resulted in significant renal recovery (creatinine 1.7 mg/dL, proteinuria 4.4 g/g). This case highlights the importance of ANCA testing in SLE patients with unexplained rapidly progressive glomerulonephritis, as early recognition of overlap syndrome carries distinct therapeutic implications, including the use of rituximab‐based regimens targeting both disease processes.

## 1. Introduction

Perinuclear antineutrophil cytoplasmic antibody (P‐ANCA) and myeloperoxidase (MPO) antibodies, typically associated with ANCA‐associated vasculitis (AAV), are detected in 15%–25% of patients with lupus nephritis [1], with MPO‐ANCA predominating in 72%–87% of cases [2]. They are markers for greater disease activity and severe kidney involvement, specifically associated with Class IV‐S lupus nephritis, glomerular necrosis, higher activity indices, worse baseline renal function, and poorer long‐term renal outcomes [3].

Rarely, true systemic lupus erythematosus (SLE)/AAV overlap syndrome occurs (estimated 2% prevalence), requiring distinct therapeutic approaches [4]. Immunosuppressive therapies, particularly belimumab, can significantly modify lupus serologies including anti‐dsDNA and complement levels, potentially complicating diagnostic interpretation [5].

We present a case of P‐ANCA/MPO positive crescentic glomerulonephritis in a patient with SLE on immunosuppressive medications without typical lupus serologies and “full house” immunofluorescence.

## 2. Case Presentation

**FIGURE 1:**
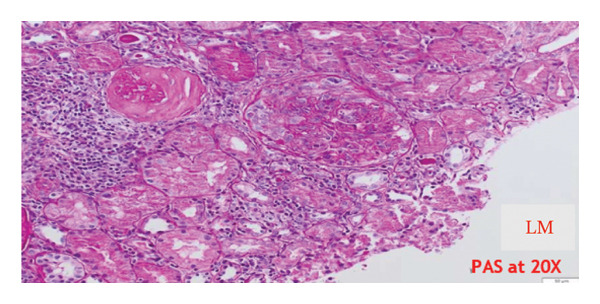
Crescentic glomerulonephritis with neutrophil‐rich infiltrates.

**FIGURE 2:**
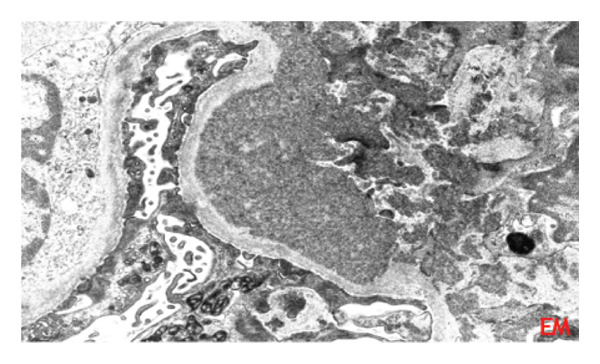
Irregular wavy GBM with focal foot process effacement. Scattered deposits.

**FIGURE 3:**
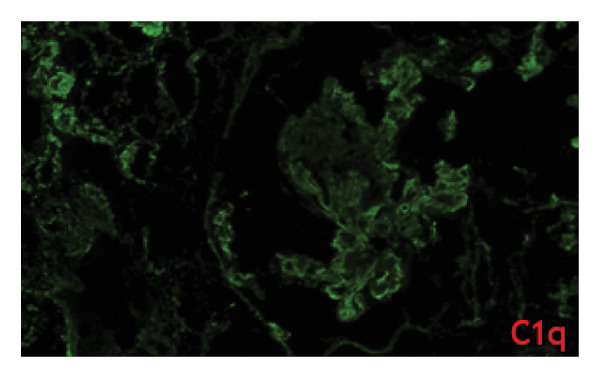
Immunofluorescence showing negative C1q staining.

**FIGURE 4:**
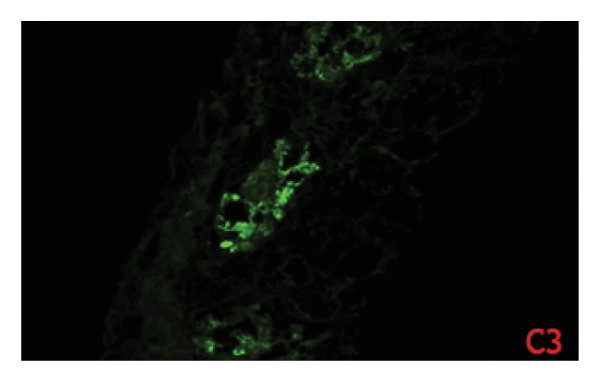
Immunofluorescence showing +3 C3 staining.

**FIGURE 5:**
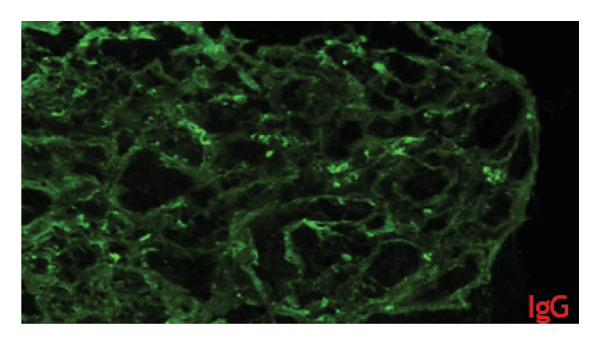
Immunofluorescence showing +2 IgG staining.

A 57‐year‐old female presented with a history of chronic kidney disease Stage II (baseline creatinine 1.1 mg/dL; estimated glomerular filtration rate [eGFR] of 65 mL/min/1.73 m^2^), proteinuria (urine protein‐to‐creatinine ratio of 379 mg/g), hypertension, diabetes mellitus, SLE (diagnosed based on a positive ANA level of 1:1280 titer, low C3, oral ulcers, and recurrent arthralgia), and APS with a history of seven miscarriages and recurrent venous thromboembolism (VTE), along with high‐titer antiphospholipid antibody positivity: anticardiolipin IgG antibody of 89 GPL units and anti‐β2‐glycoprotein I IgG antibody of 97 U/mL, on belimumab, hydroxychloroquine, and prednisone. A kidney biopsy performed at the time of SLE diagnosis had revealed diffuse diabetic glomerulosclerosis with moderate arteriosclerosis and only very rare mesangial electron‐dense deposits, without evidence of active or chronic lupus nephritis. The patient presented with M2/M3 occlusion of the middle cerebral artery requiring thrombectomy. Physical examination revealed marked bilateral lower extremity edema. Laboratory workup was remarkable for nephrotic‐range proteinuria (8.6 g/g), creatinine level (5.2 mg/dL), and normal anti‐dsDNA, C3, and C4 levels. The sepsis workup, HACEK, Bartonella PCR, HBV, HCV, HIV, and syphilis tests were all negative. The P‐ANCA titer was > 1:640 with an MPO of 12.3 U/mL. Urinalysis revealed proteinuria and hematuria with red blood cells (RBCs) greater than 100 cells per high‐power field (HPF), without dysmorphic RBCs or cellular casts, findings most consistent with traumatic hematuria secondary to Foley catheter insertion. Doppler ultrasonography of the kidney was negative for renal vein thrombosis. A kidney biopsy revealed a total of 22 glomeruli on light microscopy, of which 17 (77%) exhibited cellular crescents, consistent with crescentic glomerulonephritis. Additional findings included neutrophil‐rich glomerular infiltrates, mesangial proliferation, extensive neutrophilic interstitial inflammation with tubulitis and tubular abscesses, mild interstitial fibrosis and tubular atrophy (estimated at < 25% of the cortical area), and mild arteriosclerosis. Notably, endocapillary hypercellularity, fibrinoid necrosis, and thrombotic microangiopathy were absent (Figure 1). Immunofluorescence performed on three glomeruli from fresh frozen tissue demonstrated IgG (2+), IgM (2+), and C3 (3+) in a granular pattern, while IgA, C1q, fibrinogen, and albumin were negative. Kappa and lambda light chain staining was negative, including with pronase digestion, excluding monoclonal immunoglobulin deposition (Figures 2, 3, and 4). Electron microscopy revealed irregular wavy glomerular basement membrane contours with focal foot process effacement. Scattered electron‐dense deposits were identified in the mesangial, paramesangial, and intramembranous (within the GBM) locations, consistent with the older deposits (Figure 5). Treatment with methylprednisolone, rituximab, and a heparin bridge to warfarin improved serum creatinine to 1.7 mg/dL and proteinuria to 4.4 g/g.

## 3. Discussion

This case presents a diagnostic challenge in distinguishing between lupus nephritis and true SLE/AAV overlap syndrome. Although the absence of “full house” immunofluorescence (typically IgG, IgM, IgA, C1q, and C3) may reflect immunosuppressive effects, as belimumab reduces circulating immunoglobulins and autoantibodies potentially diminishing immune complex deposition [6, 7], several features favor true SLE/AAV overlap syndrome in this patient.

Serologically, the rapidly progressive course (serum creatinine rising from 1.1 to 5.2 mg/dL), markedly elevated P‐ANCA titer (> 1:640) with MPO positivity, and normal complement and anti‐dsDNA levels were discordant with active lupus nephritis. Histopathologically, cellular crescents in 77% of glomeruli, neutrophil‐rich infiltrates, tubulitis, and tubular abscesses are characteristic of ANCA‐mediated injury, where neutrophilic infiltration is specifically associated with glomerular necrosis and severe kidney injury [8]. The absence of C1q, a hallmark of classical complement pathway activation in immune complex‐mediated lupus nephritis, coupled with C3‐dominant staining, further supports alternative complement pathway activation increasingly recognized in ANCA‐associated glomerulonephritis [8]. On electron microscopy, the deposits were confined to mesangial, paramesangial, and intramembranous locations and characterized as older, lacking the subendothelial or subepithelial distribution typical of active immune complex–mediated disease [8]. This pattern, together with a prior biopsy showing only very rare mesangial deposits without lupus nephritis, suggests residual low‐grade immune complex accumulation from underlying SLE rather than an active immune complex–driven process. Patients with ANCA‐positive SLE with renal involvement demonstrate distinct histopathologic features, including a more frequent Class IV‐S (segmental) pattern (36% vs. 16%), glomerular necrosis (35% vs. 15%), and higher activity indices than ANCA‐negative patients [9]. MPO‐ANCA positivity, which predominates in 72%–82% of ANCA‐positive SLE cases with nephritis, is associated with higher chronicity indices, including interstitial fibrosis and tubular atrophy—findings present in this patient’s biopsy [10].

Although ANCA‐positive SLE patients present with worse baseline renal function and more severe histological activity, long‐term renal outcomes may be comparable to those of ANCA‐negative patients when treated aggressively [11]. This patient’s excellent response to rituximab plus methylprednisolone—with creatinine improving from 5.2 to 1.7 mg/dL—aligns with evidence supporting aggressive immunosuppression, including rituximab, for ANCA‐positive SLE with renal involvement.

## 4. Conclusion

This case underscores the importance of ANCA testing in SLE patients presenting with rapidly progressive glomerulonephritis. Early recognition of overlap syndrome has therapeutic implications as these patients may benefit from treatment protocols targeting both lupus and AAV, including rituximab‐based regimens. Long‐term follow‐up should include serial ANCA monitoring, as anti‐MPO ANCAs are less often associated with relapse than anti‐PR3 ANCAs are. Repeating renal biopsy may be considered if the clinical response is unsatisfactory or if there is a concern for disease transformation.

## Funding

This study was funded by Thomas Jefferson University

## Disclosure

This abstract was presented in American Journal of Kidney Disease as a Meeting Abstract [12].

## Conflicts of Interest

The authors declare no conflicts of interest.

## Data Availability

Research data are not shared.
